# Occurrence, Dietary Exposure, and Health Risk Assessment of Chlorinated Paraffins in Chicken Meat Across China

**DOI:** 10.3390/foods15020239

**Published:** 2026-01-09

**Authors:** Nan Wu, Lirong Gao, Tingting Zhou, Jiyuan Weng, Changliang Li, Wenjie Song, Yingying Zhou, Zhujun Liu, Qi Li, Yu Lu, Lei Zhang, Pingping Zhou

**Affiliations:** 1 NHC Key Laboratory of Food Safety Risk Assessment, China National Center for Food Safety Risk Assessment, Beijing 100022, China; 2 Department of Cancer Prevention and Control, National Cancer Center/National Clinical Research Center for Cancer/Cancer Hospital, Chinese Academy of Medical Sciences and Peking Union Medical College, Beijing 100021, China; 3 State Key Laboratory of Environmental Chemistry and Ecotoxicology, Research Center for Eco-Environmental Sciences, Chinese Academy of Sciences, Beijing 100085, China

**Keywords:** chlorinated paraffins, chicken meats, dietary exposure, risk assessment

## Abstract

This study systematically assessed the dietary exposure risks of short-chain and medium-chain chlorinated paraffins (SCCPs and MCCPs) through chicken consumption in China, where these persistent organic pollutants are widely produced and used. As an important component of the Chinese diet, chicken was selected as the research matrix due to its high lipid content and potential for chlorinated paraffin bio-accumulation, while available data on these contaminants in market-sold chicken remains limited. We collected 126 representative commercial chicken samples from eight major provinces and municipalities across China and conducted precise analysis using two-dimensional gas chromatography with electron capture negative ionization mass spectrometry (GC×GC-ECNI/MS). The probabilistic exposure assessment was performed through Monte Carlo simulation, and health risks were characterized using the margin of exposure (MOE) approach. The results revealed mean concentrations of 95.8 ng/g wet weight (range: 9.5–1542.4 ng/g ww) for SCCPs and 156.6 ng/g ww (range: 20.0–1517.9 ng/g ww) for MCCPs in chicken samples, with Jiangsu Province exhibiting significantly higher contamination levels compared to other regions (*p* < 0.001). The estimated mean dietary exposures through chicken consumption were 32.8 ng/kg bw/d for SCCPs and 52.6 ng/kg bw/d for MCCPs in the general Chinese population. Notably, children aged 3–6 years and the Consumer only showed the highest exposure levels. All calculated MOE values substantially exceeded the risk threshold of 1000, indicating no significant health concerns from current exposure to SCCPs and MCCPs through chicken consumption in China.

## 1. Introduction

Chlorinated paraffins (CPs), as complex mixtures of polychlorinated n-alkanes synthesized represent one of the most ubiquitous industrial chemicals in modern manufacturing [1,2]. These compounds are functionally indispensable as plasticizers in polyvinyl chloride products, flame retardants in textiles, and lubricant additives in metalworking fluids, owing to their chemical stability and cost-effectiveness [3,4]. Classification of CPs based on carbon chain length delineates short-chain CPs (SCCPs, C_10–13_), medium-chain CPs (MCCPs, C_14–17_), and long-chain CPs (LCCPs, C_>17_). China is the largest producer of CPs. From 2012 to 2020, China’s CP output increased to approximately 1 million tons per year, with nearly 1170 manufacturers [5]. SCCPs and MCCPs were listed in Annex A by the Stockholm Convention in 2017 and 2025, respectively, to restrict their use [6,7]. Despite the implementation of the phase-out of traditional persistent organic pollutants, MCCPs share structural and toxicological similarities with SCCPs, and these similarities contribute to the persistence of ecological and health risks [5,8,9]. Several studies have shown that exposure to SCCPs and MCCPs can bring many adverse effects to the human body, such as allergic diseases [10], gestational diabetes [11,12], hepatotoxicity [13], hematologic homeostasis [14], attention deficit [15]. Although the Ministry of Ecology and Environment of China officially issued a document in 2023 to ban the production, use, import, and export of SCCPs [16], the impact of these substances on ecosystems and human health persists due to their extensive historical production and use, as well as their environmental persistence and toxicity. Moreover, MCCPs have become the primary substitute for SCCPs [17]. Consequently, it is crucial to continuously monitor these chemicals, which are widely used in the environment, particularly to understand levels of human exposure.

Dietary intake constitutes the primary source of daily CPs intake, followed by dust ingestion and dermal contact [18]. Previous studies have confirmed the presence of CPs in food [19]. Krätschmer et al. identified the presence of SCCPs contamination in meat, milk, and eggs available on the German market [20]. Similarly, the Sixth Chinese Total Diet Study detected SCCPs and MCCPs in various food categories, including eggs, milk, meat, and aquatic products [6]. Furthermore, Huang et al. reported widespread contamination of both SCCPs and MCCPs in meat samples collected from 20 provinces across China [21]. The pollution level in the Chinese region is relatively high. Current evidence suggests the population may have been widely exposed to CPs through food, thus urgently demanding a comprehensive risk assessment. Previously, the European Food Safety Authority (EFSA) [22] evaluated the health risks of CPs exposure for Europeans based primarily on fish samples. However, owing to the restricted variety of samples, precise risk characterization proved unattainable, necessitating additional data from diverse food sources. In terrestrial food systems, animal-derived foods rich in lipids exhibit more significant accumulation of CPs compared to aquatic species [23]. Given its huge consumption level, poultry, especially chicken, demands priority consideration in exposure assessments. In 2020, China’s annual chicken consumption surged by 11.2%, reaching 15.5 million tons and securing the world’s second-highest ranking [24]. Chickens become highly efficient biological carriers for CPs accumulation through multiple pathways, including consumption of contaminated feed ingredients, inhalation of airborne particulate matter near industrial zones, and soil ingestion during free-range activities. Zhou et al. reported that free-range chickens in rural areas of the Tibetan Plateau can be exposed to CPs through soil, leading to accumulation in systemic tissues (SCCPs: 182~3450 ng/g lw, MCCPs: 396~7750 ng/g lw) [25]. Compared to free-range chickens, caged chickens have higher production yields but more limited farming space, with feed likely being the primary exposure source [26]. Studies have shown that the concentrations of SCCPs and MCCPs in animal feed range from 120~1700 ng/g and 6.4~260 ng/g, respectively [27]. However, to date, there is a lack of pollution data on CPs in caged chickens.

Although chicken meat accounts for a certain proportion of consumption among the Chinese population, the contamination status of CPs in chicken remains unclear. In this study, we collected chicken meat samples from eight regional markets and conducted a comprehensive analysis of the SCCPs and MCCPs content. The objectives were (1) to comprehensively investigate the contamination profiles and distribution patterns of CPs in commercially available chicken meat and (2) to rigorously evaluate the dietary exposure of Chinese residents to CPs through chicken consumption and characterize the consequent health risks.

## 2. Materials and Methods

### 2.1. Samples Collection and Food Consumption Survey

**Figure 1:**
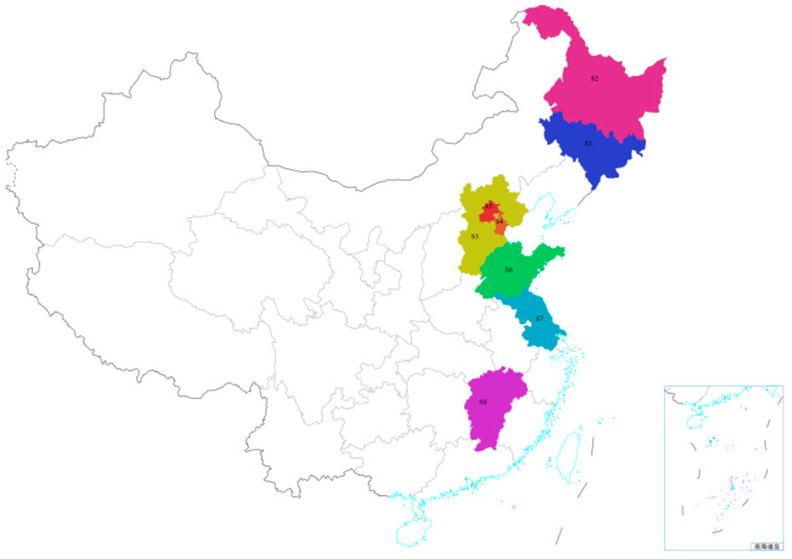
Map of eight sampling sites across China. The map was downloaded form Ministry of Natural Resources of the People’s Republic of China, and the non-English term means the South China Sea Islands.

In 2023, 126 chicken samples were collected from markets in mainland China, covering north, northeast China, and east China regions (see Figure 1). The north China regions included Hebei province (n = 15), Tianjin municipality (n = 7), and Beijing municipality (n = 10). The northeast China regions included Jilin province (n = 45) and Heilongjiang province (n = 20). The eastern China regions comprised Shandong province (n = 10), Jiangxi province (n = 11), and Jiangsu province (n = 8). The population of these regions is nearly 400 million, accounting for 28%. The output of poultry meat exceeded 14 million tons, accounting for 40% of the total output in 31 regions of the Chinese mainland [28]. All chicken samples were properly stored in a −20 °C freezer for testing after collection.

The chicken consumption data originated from the rigorous China National Food Consumption Survey conducted by the China National Center for Food Safety Risk Assessment (CFSA) spanning 2018 to 2020. This comprehensive survey utilized a sophisticated multi-stage stratified, population-proportionate cluster random sampling method, meticulously covering more than 20 provinces across mainland China. Its core methodology relied on gathering discontinuous 3-day 24 h dietary recalls from residents within the designated survey sites. Crucially, investigators mandated a minimum gap of at least three full days between consecutive 24 h recall surveys, ensuring the three survey days encompassed both weekdays and one weekend day. The initiative successfully collected vital baseline information—including gender, age, and body weight—alongside detailed daily consumption data for various chicken products from 55,700 residents. Following scrutiny, 22 individuals were excluded due to missing body weight data, leaving a robust dataset of 55,678 analyzable individuals.

### 2.2. Chemicals and Regents

The standard SCCP solutions (chlorine contents: 51.5%, 55.5%, 63.0%; 100 µg/mL, in cyclohexane) and MCCP solutions (chlorine contents: 42.0%, 52.0%, 57.0%; 100 µg/mL, in cyclohexane) were provided by Dr. Ehrenstorfer (Augsburg, Germany). The ^13^C_10_-trans-chlordane (100 μg/mL, in nonane; Cambridge Isotope Laboratories, Tewksbury, MA, USA) served as the cleanup standard. The ε-hexachlorocyclohexane solution (ε-HCH, 10 μg/mL, in cyclohexane) purchased from Dr. Ehrenstorfer (Augsburg, Germany) was used as the injection standard. Pesticide-grade n-hexane, acetone, cyclohexane, and dichloromethane were purchased from J.T. Baker (Phillipsburg, NJ, USA). Diatomaceous earth was provided by Thermo Fisher Scientific (Waltham, MA, USA). Silica gel (63–100 μm) and Florisil (60–100 mesh) were supplied by Merck KgaA (Darmstadt, Germany).

### 2.3. Sample Preparation and Analysis

The chicken meat samples were homogenized, freeze-dried, and pulverized before analysis. Approximately 5 g of the homogenized sample was extracted by accelerated solvent extraction (ASE 200, Dionex, Sunnyvale, CA, USA) with n-hexane/dichloromethane (1:1, *v*/*v*). The sample was placed in an ASE extraction cell (66 mL) with anhydrous sodium sulfate, and then 2.5 ng of ^13^C_10_-trans-chlordane was added. The specific operation method was basically consistent with the previous description [6,21]. Further purification was conducted with a multilayer composite silica gel column (packed sequentially from bottom to top with 3.00 g Florisil, 2.00 g activated silica, 5.00 g acidified silica, and 4.00 g anhydrous sodium sulfate). Before loading the sample, the multilayer composite silica gel column was pre-washed with 50 mL of n-hexane. The sample was subsequently added to the silica gel column, followed by elution with 40 mL of n-hexane, and the eluate was discarded. Then, the target compounds were eluted with 100 mL of dichloromethane/n-hexane (1:1, *v*/*v*). The collected eluate was sequentially concentrated using a rotary evaporator under reduced pressure and a nitrogen evaporator. After adding 2.5 ng of ε-HCH as an injection standard, the solution was brought to a final volume of 50 µL with cyclohexane, vortex-mixed, and subjected to instrumental analysis. A two-dimensional gas chromatography with electron capture negative ionization mass spectrometry (GC×GC-ECNI-MS; Agilent Technologies, Santa Clara, CA, USA) was used to analyze chlorinated paraffins in the samples. A total of 24 SCCPs and 24 MCCPs were detected. The specific settings of the instrument parameters have been described in our previous study [29].

### 2.4. Quality Assurance and Quality Control

Stringent quality assurance and quality control (QA/QC) protocols were implemented throughout the entire analytical workflow to ensure the reliability of the results. For the assessment of SCCPs and MCCPs contamination, a procedural blank was incorporated into each batch of twenty samples during analysis. The preparation of procedural blanks followed the identical steps applied to food samples, with the only exception that clean diatomaceous earth was substituted for the actual food matrix in the extraction stage. Results showed that most SCCP and MCCP congener groups were below the detection limit in all procedural blanks. The only exceptions were C_10_Cl_5–7_ and C_11_Cl_5–6_ congener groups, which were detected at concentrations accounting for less than 10% of the minimum quantifiable level in the actual samples. To evaluate the method reproducibility, one sample was randomly selected from each food category and analyzed in triplicate; the relative standard deviations (RSDs) of the measured concentrations were all below 10%. The method detection limit (MDL) for SCCPs was defined as the mean concentration of SCCPs detected in blanks plus three times the relative standard deviation of blank measurements, with a calculated value of 3 ng/g. For MCCPs, the MDL was determined by spiking blank samples with MCCPs at a concentration of 20 ng/g and performing seven parallel determinations; based on the relative standard deviation of these replicate analyses, the MDL for MCCPs was calculated to be 5.0 ng/g. Additionally, the recoveries of the surrogate standard (^13^C_10_-trans-chlordane) across all samples ranged from 82% to 115%.

### 2.5. Dietary Exposure of SCCPs and MCCPs from Chicken Meat

According to the probabilistic assessment method described in the World Health Organization’s publication [30], “PRINCIPLES AND METHODS FOR THE RISK ASSESSMENT OF CHEMICALS IN FOOD,” dietary exposure was calculated. The specific method involves multiplying the content of CPs in chicken by individual consumption, adjusting for individual body weight, and performing Monte Carlo simulations. The calculation is shown in the following formula:

$$\mathrm{EDI}=\frac{C\;\times \;F}{BW}$$

where *EDI* is the daily per kilogram body weight CPs exposure for a surveyed individual, *F* is the daily consumption of chicken by the surveyed subject, *C* is the concentration of CPs in chicken samples, and *BW* is the body weight of the surveyed subject.

The Margin of Exposure (MOE) approach was suitable for assessing the health risks of CPs. It was described as the ratio of a separation point to an exposure level. The ratio > 1000 suggests that no significant health concerns. This process can be described by the following formula:

$$MOE=\frac{POD}{Exp}$$

where *POD* is the point of departure, which can be represented by BMDL_10_. This study used the BMDL_10_ values for SCCPs and MCCPs recommended by EFSA, which are 2.3 mg/kg bw/d and 36 mg/kg bw/d, respectively [22].

### 2.6. Statistical Analysis

Data organization and preliminary analysis were completed using Microsoft Excel. The concentrations of SCCPs and MCCPs were expressed as mean ± standard deviation. Since the CPs concentration data did not follow a normal distribution, non-parametric estimation and Kruskal–Wallis H tests were employed to analyze their differences. The Pearson correlation method was used to compare the correlations between SCCPs and MCCPs in different regions. All the aforementioned statistical analyses were performed using R (version 4.0.3). Probabilistic exposure assessment was performed using Monte Carlo simulation (@RISK version 8.8.1) with 10,000 iterations. Concentration, consumption, and body weight inputs were modeled using fitted parametric distributions specific to each population subgroup; full details of distribution fitting, sensitivity analysis, and simulation settings are provided in the Supplementary Materials. The exposure level was expressed as the mean and 95% confidence interval (95% CI).

## 3. Results

### 3.1. Concentrations of SCCPs and MCCPs in Chicken Samples

This study reports the concentrations of SCCPs and MCCPs in chicken meat on a wet weight (ww) basis. Table 1 outlined the detection rates, mean concentrations, and concentration ranges of SCCPs and MCCPs in chicken samples from various regions. Both contaminants were found in all regional samples, with overall mean concentrations of 95.8 ng/g ww (ranging from 9.5 to 1542.4 ng/g ww) for SCCPs and 156.6 ng/g ww (ranging from 20.0 to 1517.9 ng/g ww) for MCCPs. The regional mean concentrations of SCCPs in chicken samples from Jilin, Heilongjiang, Hebei, Tianjin, Beijing, Shandong, Jiangxi, and Jiangsu ranged from 21.5 to 497.6 ng/g ww. Corresponding MCCP concentrations ranged from 29.9 to 299.2 ng/g ww, respectively. Comparative analysis indicated that chicken samples from Jiangsu Province had significantly higher concentrations of SCCPs and MCCPs compared to other regions (*p* < 0.001). Additionally, geographic classification revealed mean concentrations of 156.2 ng/g ww (SCCPs) and 122.6 ng/g ww (MCCPs) in East China regions (Jiangxi, Jiangsu, Shandong), 73.4 ng/g ww (SCCPs) and 135.9 ng/g ww (MCCPs) in North China regions (Hebei, Tianjin, Beijing), contrasted with 79.8 ng/g ww (SCCPs) and 182.0 ng/g ww (MCCPs) in Northeast China regions (Jilin, Heilongjiang). We applied a multiple-comparison adjustment to the post hoc analyses. Specifically, *p*-values from the pairwise Wilcoxon tests were adjusted using the Benjamini–Hochberg false discovery rate (BH-FDR) procedure, and the results revealed that the differences among regions remained statistically significant (details are provided in Tables S2 and S3).

### 3.2. Composition Profiles of SCCPs and MCCPs in Chicken Samples

**Figure 2:**
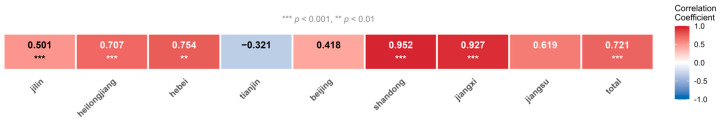
Spearman correlations between SCCPs and MCCPs in different regions.

**Figure 3:**
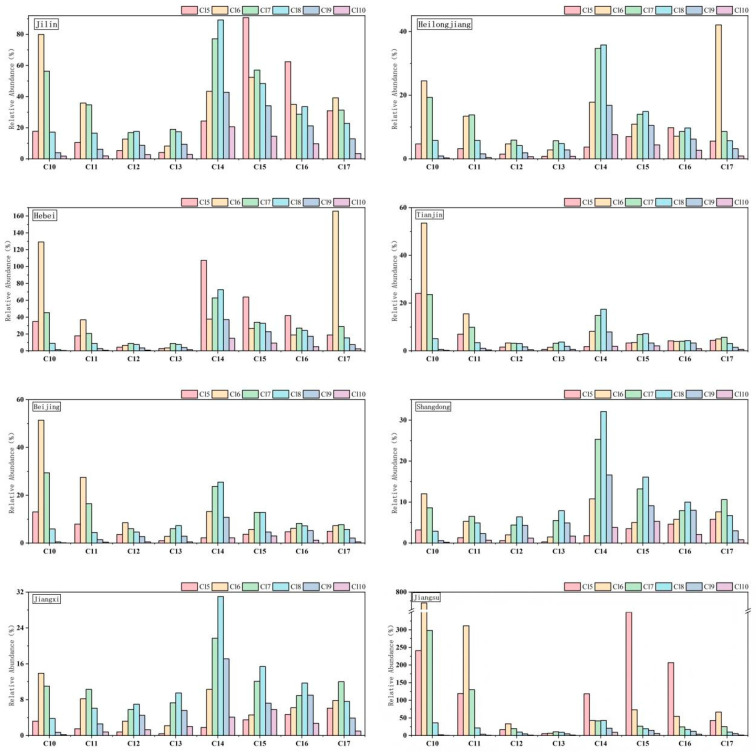
The concentrations of SCCPs and MCCPs congener group (ng/g) in chicken meat.

We analyzed the correlation between SCCPs and MCCPs in chicken samples from different regions, as depicted in Figure 2. Significant positive correlations were observed in samples from Jilin (r = 0.501, CI: 0.22–0.71, *p* < 0.001), Heilongjiang (r = 0.707, CI: 0.41–0.85, *p* < 0.001), Hebei (r = 0.754, CI: 0.28–0.97, *p* = 0.002), Jiangxi (r = 0.927, CI: 0.72–1.00, *p* < 0.001), and Shandong (r=0.952, CI: 0.69–1.00, *p* < 0.001). Additionally, a significant positive correlation was revealed between SCCPs and MCCPs in total chicken samples (r = 0.721, CI: 0.61–0.80, *p* < 0.001). This study analyzed 24 SCCP and 24 MCCP groups extracted from the chicken samples; the concentration data for each group are presented in Figure 3. Regarding SCCPs, the congener distribution patterns were similar across regions, with C_10-11_Cl_6-7_ being the predominant carbon-chlorine congener group. Based on carbon chain length, C_10_-CPs were the most abundant carbon homologues, accounting for 29~63% of the total SCCPs in chicken samples, followed by C_11_-CPs (22~29%). In terms of chlorination degree, Cl_6_-CPs and C_7_-CPs contributed the most to SCCPs, constituting 23~55% and 22~34%, respectively. Unlike other regions, Cl_8_CPs accounted for 25% of SCCPs in Shandong samples, exceeding the contribution of Cl_7_-CPs. For MCCPs, the contribution rates varied regionally. MCCPs constituted over 60% of the total CPs in samples from Jilin, Heilongjiang, Hebei, Shandong, and Jiangxi. In contrast, MCCPs accounted for 41%, 47%, and 37% of total CPs in samples from Tianjin, Beijing, and Jiangsu, respectively, which is lower than the contribution from SCCPs. Unlike SCCPs, the distribution profiles of MCCP congeners exhibited differences. In the chicken samples from the mentioned regions, the carbon homologue abundances were broadly similar, with C_14-15_-CPs contributing 58–66% to MCCP concentrations. The distribution of chlorine homologue groups showed slight regional variations: Cl_5-6_-CPs were the predominant congener groups in Jilin, Hebei, and Jiangsu, with contributions of 41%, 54%, and 77%, respectively. In other regions, Cl_7-8_-CPs were predominant, contributing 46~57%. Notably, the contribution of Cl_5_-CPs in Jiangsu samples was significantly higher (58%) than in other regions.

### 3.3. Human Exposure Assessment

This study stratified 55,678 individuals into eight subgroups by age and sex (Male 3–6 years, Male 7–12 years, Male 13–17 years, Female 13–17 years, Male 18–59 years, Female 18–59 years, Male ≥ 60 years, Female ≥ 60 years), and calculated the daily consumption amounts for each subgroup. We also defined a consumer-only group, whose members consumed chicken daily (see Table 2). The average chicken consumption for the entire population was 16.8 g, with a maximum consumption of 733.3 g. The daily average chicken consumption ranges for the eight subgroups were 11.5~22.0 g, respectively. Compared to the other subgroups, the consumer group exhibited higher average consumption and 95th percentile (P95) consumption values, at 54.7 g and 133.3 g, respectively. Based on individual consumption data and body weight data, we estimated the average exposure levels to SCCPs and MCCPs across the different subgroups. As shown in Table 3, this study estimated the average population-level chicken-derived SCCP and MCCP exposures in China to be 32.8 (95% CI: 2.4) ng/kg bw/d and 52.6 (95% CI: 3.5) ng/kg bw/d, respectively. The exposure levels for high consumers were 106.4 ng/kg bw/d and 159.8 ng/kg bw/d, respectively. The average SCCP and MCCP exposure levels across the gender-age subgroups ranged from 22.3 (95% CI: 0.5) to 89.0 (95% CI: 1.8) ng/kg bw/d and 33.2 (95% CI: 0.7) to 132.6 (95% CI: 2.7) ng/kg bw/d, respectively. Among the age-gender subgroups, the 3–6 year age group exhibited the highest average SCCP and MCCP exposures, followed by the 7–12 year age group, with exposures of 66.2 (95% CI: 51.4) ng/kg bw/d and 99.4 (95% CI: 2.1) ng/kg bw/d, respectively. Within the consumer only, the average SCCP and MCCP exposure levels were 93.9 (95% CI: 22.0) ng/kg bw/d and 153.2 (95% CI: 31.1) ng/kg bw/d, respectively. Regional variations in chlorinated paraffin contamination significantly influence population exposure levels. Therefore, we further analyzed exposure levels across different regions (see Table 3). For SCCPs, the highest per capita exposure was observed in Jiangsu and the lowest in Shandong, with values of 159.1 (95% CI: 13.2) ng/kg bw/d and 6.9 (95% CI: 0.6) ng/kg bw/d, respectively. For MCCPs, the highest per capita exposure was found in Jiangsu and the lowest in Tianjin, at 84.4 (95% CI: 7.2) ng/kg bw/d and 4.2 (95% CI: 3.8) ng/kg bw/d, respectively. The average SCCP exposure levels in the eastern China regions were higher than those in north China and northeast China regions, while the average MCCP exposure levels in east China regions were lower than those in north China and northeast China regions. The Contribution to Variance metric in @RISK was used for sensitivity analysis, and the results showed that dietary exposure variability was mainly driven by CP concentrations (16.8% for SCCPs and 2.3% for MCCPs), followed by consumption (2.2% and 0.6%, respectively). These results indicate that the concentration of CPs is the primary determinant of overall exposure level (details are provided in Figures S5–S8).

**Figure 4:**
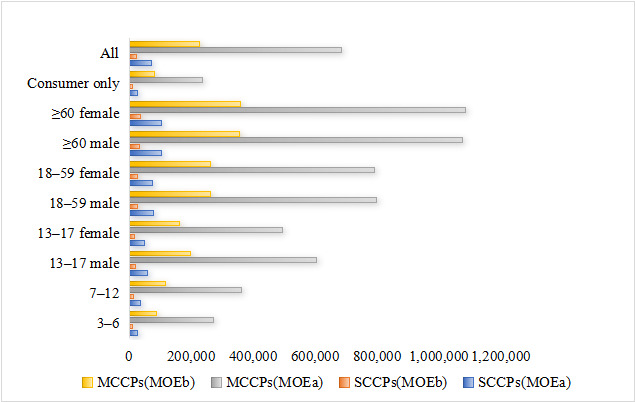
Health risk attributed to chlorinated paraffins in the whole population and in different sex-age groups; MOE^a^ for average daily intake; MOE^b^ for P95 daily intake.

**Figure 5:**
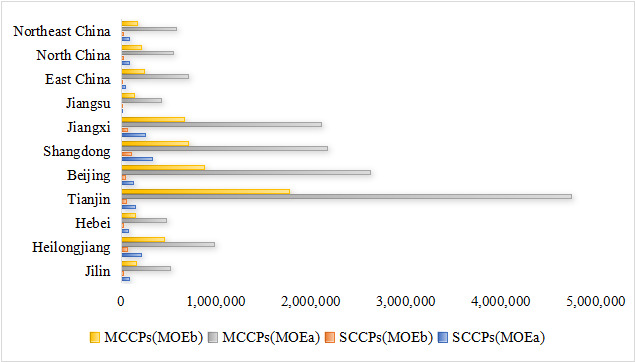
Health risk attributed to chlorinated paraffins in the whole population from different regions; MOE^a^ for average daily intake; MOE^b^ for P95 daily intake.

The health risk assessment employed two distinct Margin of Exposure (MOE) values using the corrected units for consistency. The MOE based on the mean exposure (MOE^a^) was designated for the general population, while that based on the 95th percentile exposure (MOE^b^) was used for high consumers. As illustrated in Figure 4, the calculated MOE^a^ values for SCCPs exposure across populations of all ages and genders ranged from 25,843 to 103,139, while MOE^b^ values ranged from 8358 to 33,675. For MCCPs, the MOE^a^ values spanned from 271,493 to 1,084,337, with MOE^b^ values ranging between 87,379 and 357,143. Regional variations in health risks were also evaluated. Figure 5 demonstrates that population-specific MOE^a^ values for SCCPs exposure varied from 14,456 to 333,333 across different regions, with corresponding MOE^b^ values ranging from 4736 to 107981. Similarly, for MCCPs exposure, regional MOE^a^ values extended from 426,540 to 4,736,842, while MOE^b^ values ranged between 137,562 and 1,773,399. Notably, all calculated MOE values in this study exceeded the safety threshold of 1000, indicating no significant health concerns under current exposure levels.

## 4. Discussion

Chlorinated paraffins, as a class of industrial products with extensive applications, are produced and used in large quantities. However, it cannot be overlooked that, as persistent organic pollutants, chlorinated paraffins pose a series of adverse effects on the environment and human health. Currently, the impacts of these environmental chlorinated paraffins on human health have not been widely studied, nor is human exposure clearly understood. Against this backdrop, we conducted a health assessment of human exposure to chlorinated paraffins based on chicken consumption. In this study, we analyzed the concentrations and contamination characteristics of SCCPs and MCCPs in chicken samples from eight provinces/municipalities in mainland China and further assessed the dietary risks to the Chinese population. SCCPs and MCCPs were detected in all chicken samples, with average concentrations of 95.8 ng/g ww and 156.6 ng/g ww, respectively. A 2018 study by Huang H et al. [21] in Mainland China reported SCCP and MCCP concentrations in meat at 129 ng/g ww and 5.7 ng/g ww, respectively. Cui et al. [6] found in 2020 that meat from southern China contained SCCPs and MCCPs at 69 ng/g ww and 63 ng/g ww, respectively. The average concentrations of SCCPs and MCCPs in meat samples collected by Ding et al. [31] from the Shandong region were 25.3 ng/g ww and 14.2 ng/g ww, respectively. Among these, SCCPs were consistent with our reported data from the same region, while MCCPs exhibited lower concentrations. These discrepancies may stem from China’s regulatory measures on SCCPs being offset by historical mass production, leading to environmental accumulation and subsequent transfer into organisms, coupled with increasing MCCP production as substitute products, reflected in the rising MCCP contamination levels year by year. Regional variations in SCCPs and MCCPs were also observed: chicken samples from Jiangsu exhibited significantly elevated SCCP and MCCP levels compared to other regions, followed by the Hebei region. Xia et al. revealed that concentrations of chlorinated paraffins in human breast milk significantly exceeded those in other urban centers within Hebei Province’s urban centers [32]. This regional disparity is relatively consistent with the CPs production situation in various provinces of China [17,33]. In this study, although the concentrations of SCCPs and MCCPs in chicken meat samples from different provinces varied, the MCCP content in all provinces was not lower than the SCCP content, except for Jiangsu. A possible explanation for this particularity is that Jiangsu is located in the Yangtze River Delta region of China, an area with a dense population of Homo sapiens and strong industrial activity, resulting in high SCCP emissions [34].

Correlation analysis revealed that SCCPs and MCCPs in chicken meat may have the same source [31,35]. Further studies found that the distribution patterns of SCCPs congeners were similar across different regions, with C_10–11_Cl_6–7_ being the predominant carbon-chlorine congener group, which is consistent with findings from Chinese breast milk research [32], the Sixth Chinese Total Diet Study [6], and Huang et al.’s study [21]. The distribution of MCCP chlorinated congener groups exhibits slight regional variations. In Jilin, Hebei, and Jiangsu provinces, Cl_5–6_-CPs predominate, while Cl_7–8_-CPs are more prevalent in other regions. Notably, the contribution of Cl_5_-CPs in Jiangsu samples is significantly higher than in other areas. The discrepancies in the contamination levels of these CP congeners are likely attributable to variations in the production and application of technical CP mixtures across different provinces in China [36].

Diet represents the primary pathway for human exposure to chlorinated paraffins [37]. This study specifically assessed chlorinated paraffin intake via chicken consumption across diverse age and gender groups in China. Huang et al. [21] documented SCCPs exposure from meat consumption in the Chinese population, ranging from 20~560 ng/kg bw/d, while MCCPs exposure was significantly lower, at 0.3~31 ng/kg bw/d. These MCCP findings are strikingly lower than our own results. In stark contrast, the Sixth Chinese Total Diet Study [6] reported considerably higher SCCP and MCCP levels in meat, at 110 ng/kg bw/d and 100 ng/kg bw/d, respectively. A plausible explanation is that their reported meat consumption data included pork, which accounts for over 50% of total meat intake. This substantial level of pork consumption compensates for the lower concentrations of SCCPs and MCCPs, ultimately resulting in a higher overall exposure estimate. Assuming that the contamination levels in other meat types are of the same order of magnitude as those in this study, and based on the estimate that poultry constitutes about 25% of total meat consumption in China [38], the resulting MOEs corresponding to the total dietary exposure of SCCPs and MCCPs from all meat sources were all below 1000. Although we have addressed the contamination characteristics of CPs in chicken and the corresponding health risk data, this study still has some limitations: 1. The cooking method of chicken (such as frying) has a strong positive correlation with the final intake of the population, and the influence of processing methods needs to be further considered; 2. For the Chinese population, grains, vegetables, pork, eggs and milk are the primary food sources, so the impact of these foodstuffs should be considered in future research to further conduct risk assessments. 3. We did not detect LCCPs in the study, although the research on LCCPs was not as in-depth as that on SCCPs and MCCPs, the health hazards they brings as persistent organic pollutants still need to be taken into account.

## 5. Conclusions

The research analyzed contamination patterns and homologue composition in chicken meat; the results indicate that chicken consumption poses no significant health concern to the Chinese population based on chicken-derived exposure only. Continued monitoring is recommended due to the persistent use of MCCPs and LCCPs.

## Figures and Tables

**Table 1:** SCCPs and MCCPs concentrations (ng/g ww) in chicken meat samples.

Sample	Regions	Concentrations of SCCPs			Concentrations of MCCPs			*p*-Value	
		Samples Detected	Mean	Range	Samples Detected	Mean	Range	SCCPs	MCCPs
Chicken meat	Jilin	45 (100%)	99.7	26.6~470.2	45 (100%)	228.4	86.3~910.7	<0.001 ^a^	
	Heilongjiang	20 (100%)	35.0	16.7~82.3	20 (100%)	77.6	20.8~161.6		
	Hebei	15 (100%)	102.7	9.5~374.8	15 (100%)	246.4	32.8~1517.9		
	Tianjin	7 (100%)	42.6	23.0~63.3	7 (100%)	29.9	20.0~57.6		
	Beijing	10 (100%)	51.2	16.8~135.5	10 (100%)	44.3	20.5~94.5		
	Shandong	10 (100%)	21.5	14.4~30.6	10 (100%)	52.0	29.7~73.6		
	Jiangxi	11 (100%)	30.4	12.2~125.9	11 (100%)	58.3	23.4~136.7		
	Jiangsu	8 (100%)	497.6	32.1~1542.4	8 (100%)	299.2	49.3~990.8		
	East China	29 (100%)	156.2	12.2~1542.4	29 (100%)	122.6	23.4~990.8	0.001 ^b^	<0.001 ^c^
	North China	32 (100%)	73.4	9.5~374.8	32 (100%)	135.9	20.0~1517.9		
	Northeast China	65 (100%)	79.8	16.7~470.2	65 (100%)	182.0	20.8~910.7		
Total		126 (100%)	95.8	9.5~1542.4	126 (100%)	156.6	20.0~1517.9		

a: Kruskal–Wallis test for 8 areas on levels of SCCPs and MCCPs, b: Kruskal–Wallis test for 3 regions on levels of SCCPs, c: Kruskal–Wallis test for 3 regions on levels of MCCPs.

**Table 2:** Chicken meat-derived paraffin chloride exposure levels (ng/kg bw/d) in the whole population and in different sex-age groups.

Group	SCCPs		MCCPs	
	Mean (95% CI)	P95	Mean (95% CI)	P95
3–6	89.0 ± 1.8	275.2	132.6 ± 2.7	412.0
7–12	66.2 ± 1.4	206.7	99.4 ± 2.1	313.4
13–17 male	39.8 ± 0.8	122.6	59.6 ± 1.3	183.0
13–17 female	49.1 ± 1.0	150.0	73.1 ± 1.5	222.7
18–59 male	30.2 ± 0.6	91.8	45.2 ± 0.9	137.5
18–59 female	30.6 ± 0.6	92.1	45.5 ± 0.9	137.6
≥60 male	22.5 ± 0.5	70.0	33.5 ± 0.7	101.7
≥60 female	22.3 ± 0.5	68.3	33.2 ± 0.7	100.8
Consumer only	93.9 ± 22.0	290.7	153.2 ± 31.1	450.9
All	32.8 ± 2.4	106.4	52.6 ± 3.5	159.8

CI: Confidence Interval.

**Table 3:** Chicken meat-derived chlorinated paraffin exposure levels (ng/kg bw/d)for the whole population from different regions.

Regions	SCCPs		MCCPs	
	Mean (95% CI)	P95	Mean (95% CI)	P95
Jilin	27.0 ± 4.7	92.2	68.8 ± 6.5	225.3
Heilongjiang	10.7 ± 0.4	33.7	36.5 ± 22.3	78.2
Hebei	29.7 ± 6.4	97.4	75.3 ± 4.4	241.1
Tianjin	15.6 ± 4.0	42.1	7.6 ± 2.0	20.3
Beijing	17.9 ± 2.8	53.1	13.7 ± 2.0	41.2
Shangdong	6.9 ± 0.6	21.3	16.6 ± 1.5	50.9
Jiangxi	8.8 ± 2.3	33.1	17.1 ± 2.0	53.9
Jiangsu	159.1 ± 13.2	485.6	84.4 ± 7.2	261.7
East China	50.6 ± 8.5	160.4	50.9 ± 11.9	146.2
North China	25.7 ± 5.5	83.2	65.3 ± 47.3	167.3
Northeast China	27.7 ± 4.5	92.9	62.1 ± 11.4	204.2

CI: Confidence Interval.

## Data Availability

The original contributions presented in this study are included in the article/Supplementary Materials. Further inquiries can be directed to the corresponding authors.

## Supplementary Materials

The following supporting information can be downloaded at: https://www.mdpi.com/article/10.3390/foods15020239/s1, Table S1: Fitted distribution situation; Table S2: Post-hoc comparison results for SCCP; Table S3: Post-hoc comparison results for MCCP; Table S4: Correlation analysis results; Table S5: The concentrations of SCCPs congener group (ng/g ww) in chicken meat; Table S6: The concentrations of MCCPs congener group (ng/g ww) in chicken meat Jilin; Figure S1: A Total Ion Chromatogram (TIC) of a SCCPs; Figure S2: ECNI-MS spectrum of C_10_C_l6_ (SCCP); Figure S3: A Total Ion Chromatogram (TIC) of a MCCPs; Figure S4: ECNI-MS spectrum of C_14_C_l8_ (SCCP); Figure S5: Sensitivity analysis based on whole population exposure to SCCPs; Figure S6: Sensitivity analysis based on whole population exposure to MCCPs; Figure S7: Sensitivity analysis based on consume only exposure to SCCPs; Figure S8: Sensitivity analysis based on consume only exposure to MCCPs.
