# Supplementary material for: Occurrence, Dietary Exposure, and Health Risk Assessment of Chlorinated Paraffins in Chicken Meat Across China
Source: Foods. 2026 Jan 9;15(2):239. doi: 10.3390/foods15020239 (PMC12839867; doi:10.3390/foods15020239)
Supplement: Supplementary file 1 [file foods-15-00239-s001.zip › foods-4052341-supplementary.pdf]

Text S1 Settings of @risk

iteration：10000　simulation：1

Table S1 Fitted distribution situation

	Concentrations		Consumption	Weight
	SCCP	MCCP		
Whole population	Pearson5	Lognorm	Expon	Laplace
3-6	Pearson5	Lognorm	Expon	Loglogistic
7-12	Pearson5	Lognorm	Expon	Pearson5
13-17 M	Pearson5	Lognorm	Expon	Loglogistic
13-17 F	Pearson5	Lognorm	Expon	Loglogistic
18-59 M	Pearson5	Lognorm	Expon	Loglogistic
18-59 F	Pearson5	Lognorm	Expon	Loglogistic
60 M	Pearson5	Lognorm	Expon	Loglogistic
60 F	Pearson5	Lognorm	Expon	Loglogistic
Consumer only	Pearson5	Lognorm	Invgauss	Laplace

Text S2 The data did not meet the normality assumption; therefore, the Kruskal-Wallis nonparametric test was employed. The results indicated statistically significant differences among the regions ($p < 0.001$). Post-hoc comparisons were subsequently conducted as below

Table S2 Post-hoc comparison results for SCCP

Groups	Adjust p-value
East China VS North China	0.0465

East China VS Northeast China	0.0022
North China VS Northeast China	0.0465

Continued

**Province_1**	**Province_2**	**Adjust p-value**
jilin	heilongjiang	<0.001
shandong	jilin	<0.001
jilin	beijing	<0.001
jilin	jiangxi	<0.001
tianjin	jilin	<0.001
tianjin	hebei	<0.001
hebei	beijing	0.001
jiangsu	beijing	0.001
tianjin	heilongjiang	0.001
jiangxi	hebei	0.001
tianjin	jiangsu	0.002
shandong	hebei	0.002
jiangsu	heilongjiang	0.002
shandong	jiangsu	0.003
jiangxi	jiangsu	0.003
heilongjiang	hebei	0.006
tianjin	shandong	0.016
heilongjiang	beijing	0.020
tianjin	jiangxi	0.039

Table S3 Post-hoc comparison results for MCCP

Groups	Adjust p-value
East China VS North China	0.00016
East China VS Northeast China	0.00016
North China VS Northeast China	0.56123

Continued

Province_1	Province_2	Adjust p-value
shandong	jilin	<0.001
jilin	heilongjiang	<0.001
jilin	jiangxi	<0.001
shandong	jiangsu	<0.001
jiangxi	jiangsu	0.003
tianjin	jilin	0.003
tianjin	shandong	0.003
jiangsu	heilongjiang	0.007
shandong	hebei	0.017
shandong	beijing	0.019
jilin	beijing	0.024
jiangsu	beijing	0.044
jiangxi	beijing	0.044

Text S3 Spearman's rank correlation had already been applied in the original analysis, but this was not clearly described in the Methods section. We have now clarified the statistical method used and supplemented the results by reporting the 95% confidence intervals.

Table S4 Correlation analysis results

Province	Correlation	P_value
jilin	0.50 (0.22, 0.71)	0.001
heilongjiang	0.71 (0.41, 0.85)	0.001
hebei	0.75 (0.28, 0.97)	0.002
jiangsu	0.62 (-0.20, 1.00)	0.115
beijing	0.42 (-0.36, 0.89)	0.232
tianjin	-0.32 (-1.00, 1.00)	0.498
jiangxi	0.93 (0.72, 1.00)	< 0.001
shandong	0.95 (0.69, 1.00)	< 0.001
total	0.72 (0.61, 0.80)	< 0.001

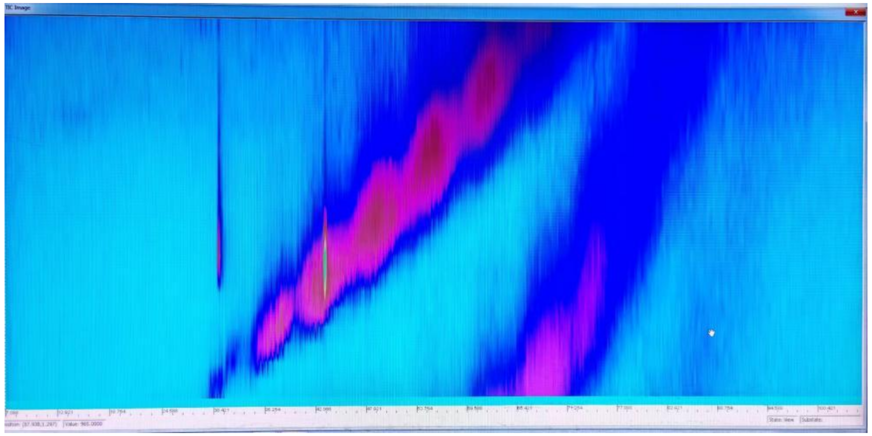

Figure S1 A Total Ion Chromatogram (TIC) of a SCCPs

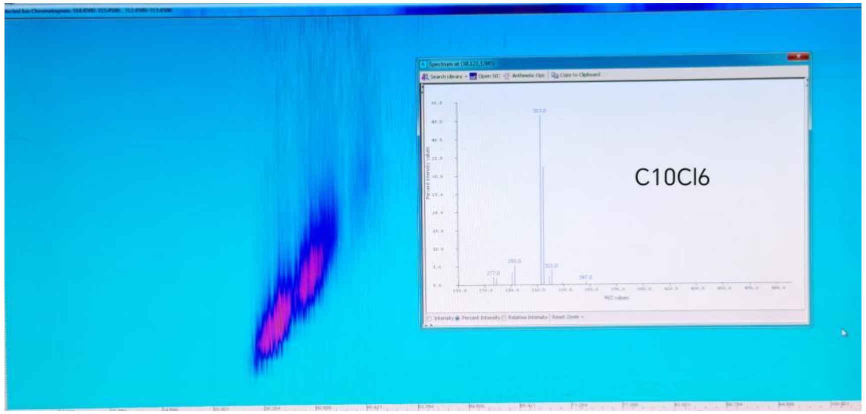

Figure S2 ECNI-MS spectrum of $C_{10}C_{l6}$ (SCCP)

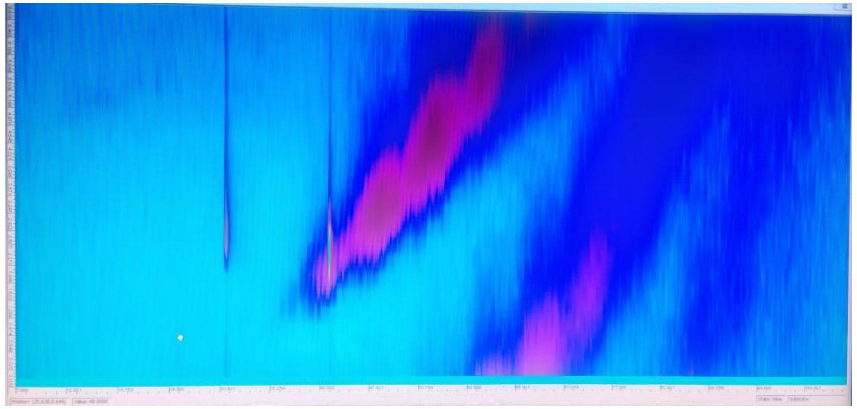

Figure S3 A Total Ion Chromatogram (TIC) of a MCCPs

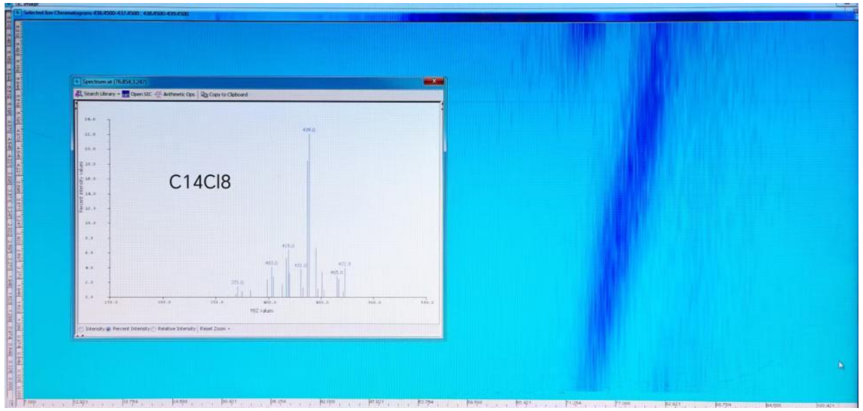

Figure S4 ECNI-MS spectrum of $C_{14}Cl_8$ (SCCP)

Table S5 The concentrations of SCCPs congener group (ng/g ww) in chicken meat

	Jilin	Heilongjiang	Hebei	Tianjin	Beijing	Shangdong	Jiangxi	Jiangsu
$C_{10}Cl_5$	17.7	4.7	34.8	24.1	13.0	3.2	3.2	240.9
$C_{10}C_{l6}$	79.9	24.5	129.2	53.5	51.4	12.0	13.9	769.2
$C_{10}Cl_7$	56.3	19.3	45.3	23.6	29.4	8.6	11.0	298.1
$C_{10}Cl_8$	17.2	5.8	8.9	5.1	5.9	2.9	3.8	36.0
$C_{10}Cl_9$	3.9	0.9	1.4	0.6	0.5	0.6	0.7	2.0
$C_{10}Cl_{10}$	1.8	0.3	0.4	0.2	0.1	0.2	0.2	0.4
$C_{11}Cl_5$	10.5	3.2	17.8	7.0	7.9	1.3	1.5	119.1
$C_{11}Cl_6$	35.9	13.4	36.9	15.5	27.5	5.3	8.2	311.3
$C_{11}Cl_7$	34.7	13.8	20.6	9.9	16.5	6.5	10.3	130.1
$C_{11}Cl_8$	16.5	5.8	8.8	3.4	4.4	4.9	6.1	21.4
$C_{11}Cl_9$	6.2	1.6	2.7	1.1	1.4	2.3	2.6	3.7
$C_{11}Cl_{10}$	1.9	0.4	0.7	0.4	0.4	0.7	0.8	0.7
$C_{12}Cl_5$	5.4	1.5	4.4	1.6	3.6	0.6	0.8	16.7
$C_{12}Cl_6$	12.7	4.7	6.6	3.3	8.5	2.0	3.2	33.2
$C_{12}Cl_7$	16.9	5.9	8.8	3.2	6.0	4.4	5.8	19.6
$C_{12}Cl_8$	17.6	4.2	7.5	3.1	4.6	6.4	7.0	9.7
$C_{12}Cl_9$	8.7	1.9	3.5	1.7	2.7	4.3	4.5	4.5
$C_{12}Cl_{10}$	2.7	0.7	0.9	0.5	0.5	1.2	1.3	1.1
$C_{13}Cl_5$	4.1	0.8	2.7	0.6	1.0	0.3	0.4	5.2
$C_{13}Cl_6$	8.3	2.8	3.5	1.5	2.8	1.5	2.2	6.6
$C_{13}Cl_7$	19.0	5.7	8.7	3.2	6.0	5.5	7.3	10.6
$C_{13}Cl_8$	17.4	4.8	7.5	3.7	7.3	7.9	9.5	9.0

$C_{13}Cl_9$	9.4	2.8	3.9	1.9	2.9	4.9	5.6	4.8
$C_{13}Cl_{10}$	2.8	0.8	1.4	0.6	0.5	1.7	2.0	1.3

Table S6 The concentrations of MCCPs congener group (ng/g ww) in chicken meat

	Jilin	Heilongjiang	Hebei	Tianjin	Beijing	Shangdong	Jiangxi	Jiangsu
$C_{14}Cl_5$	24.3	3.7	107.4	1.8	2.2	1.8	1.8	118.3
$C_{14}Cl_6$	43.4	17.8	37.7	8.2	13.2	10.8	10.3	42.7
$C_{14}Cl_7$	77.1	34.7	62.8	14.8	23.7	25.3	21.7	41.5
$C_{14}Cl_8$	89.2	35.8	72.6	17.4	25.5	32.1	31.0	42.8
$C_{14}Cl_9$	42.8	16.8	37.1	7.9	10.8	16.6	17.1	20.6
$C_{14}Cl_{10}$	20.6	7.6	14.9	1.9	2.2	3.8	4.1	9.0
$C_{15}Cl_5$	90.7	7.0	63.9	3.3	3.7	3.5	3.5	349.8
$C_{15}Cl_6$	52.4	10.9	26.6	3.5	5.7	5.0	4.6	73.0
$C_{15}Cl_7$	57.0	14.0	34.0	6.9	12.8	13.2	12.1	26.3
$C_{15}Cl_8$	48.4	14.9	32.7	7.2	12.8	16.1	15.4	19.3
$C_{15}Cl_9$	34.1	10.5	22.8	3.3	4.6	9.1	7.2	14.6
$C_{15}Cl_{10}$	14.5	4.4	9.2	2.1	3.0	5.3	5.8	6.0
$C_{16}Cl_5$	62.4	9.8	41.8	4.2	4.7	4.6	4.7	206.8
$C_{16}Cl_6$	35.0	7.1	18.9	3.9	6.2	5.8	6.2	54.5
$C_{16}Cl_7$	28.7	8.6	27.0	4.0	8.2	7.9	8.9	24.6
$C_{16}Cl_8$	33.6	9.7	24.2	4.3	7.2	10.0	11.7	16.8
$C_{16}Cl_9$	21.2	6.2	17.4	3.3	5.2	8.0	9.0	11.9
$C_{16}Cl_{10}$	9.7	2.7	5.0	0.9	1.2	2.1	2.7	3.6
$C_{17}Cl_5$	30.9	5.6	18.9	4.4	4.9	5.8	6.1	42.4
$C_{17}Cl_6$	39.2	42.1	165.8	5.0	7.3	7.6	7.8	66.6

$C_{17}Cl_7$	31.3	8.6	28.9	5.7	7.7	10.6	12.0	25.1
$C_{17}Cl_8$	22.8	5.7	15.4	3.1	5.7	6.7	7.6	9.7
$C_{17}Cl_9$	12.9	3.2	7.4	1.5	2.1	3.0	3.9	5.4
$C_{17}Cl_{10}$	3.4	0.9	2.4	0.6	0.5	0.8	1.0	1.4

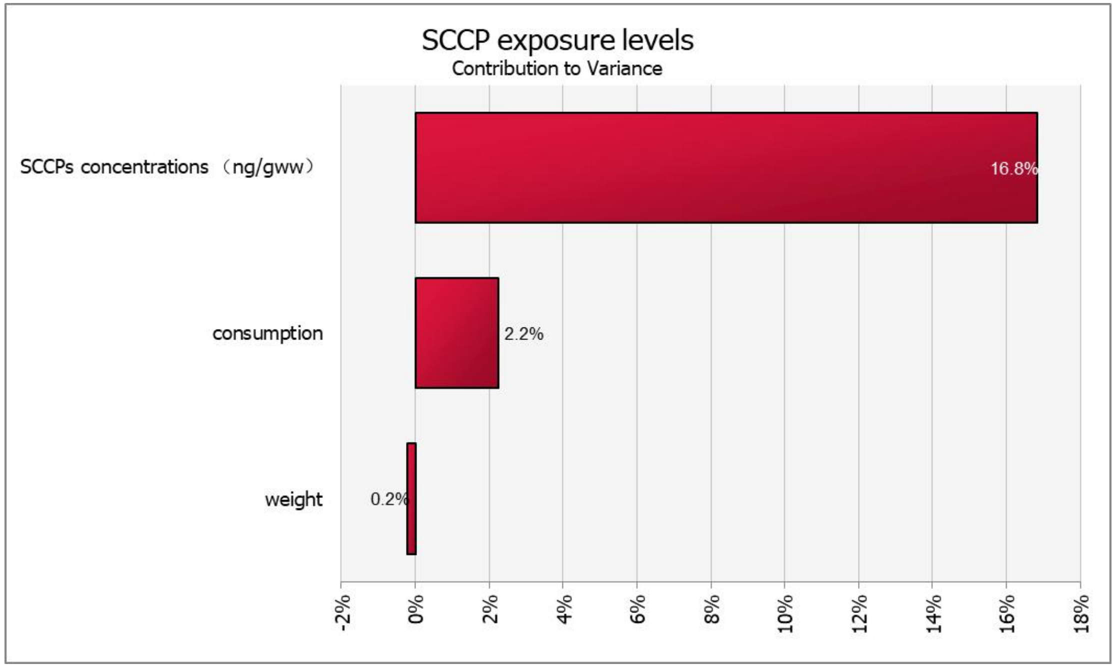

Figure S5 Sensitivity analysis based on whole population exposure to SCCPs

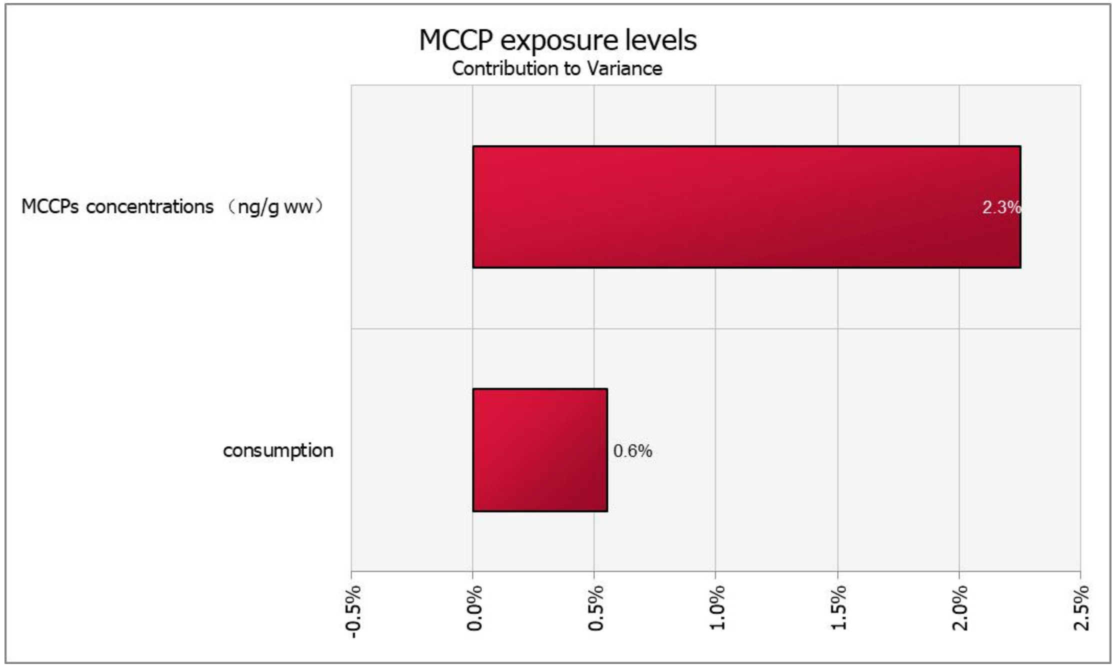

Figure S6 Sensitivity analysis based on whole population exposure to MCCPs

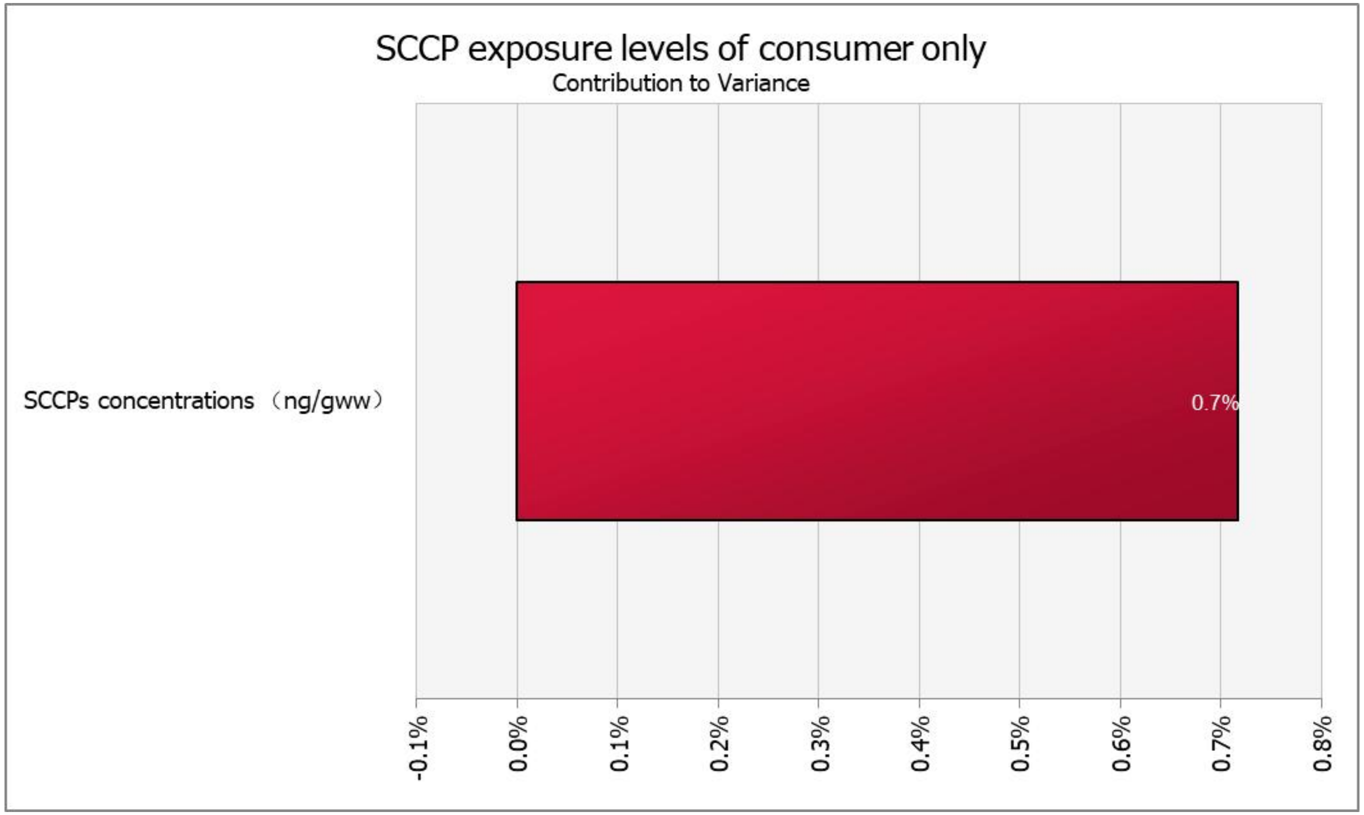

Figure S7 Sensitivity analysis based on consume only exposure to SCCPs

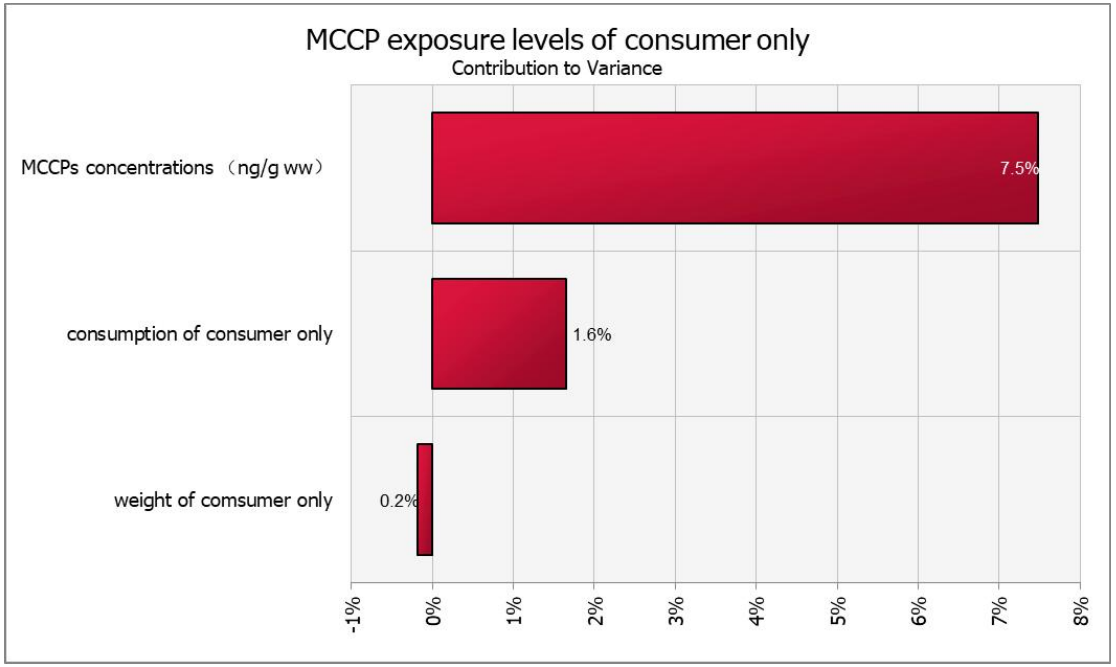

Figure S8 Sensitivity analysis based on consume only exposure to MCCPs
